# Resting State Functional Connectivity Associated With Sahaja Yoga Meditation

**DOI:** 10.3389/fnhum.2021.614882

**Published:** 2021-03-16

**Authors:** Alfonso Barrós-Loscertales, Sergio Elías Hernández, Yaqiong Xiao, José Luis González-Mora, Katya Rubia

**Affiliations:** ^1^Department of Psicología Básica, Clínica y Psicobiología, Universitat Jaume I, Castellón, Spain; ^2^Departamento de Ingeniería Industrial, Universidad de La Laguna, Tenerife, Spain; ^3^Autism Center of Excellence, Department of Neurosciences, University of California, San Diego, San Diego, CA, United States; ^4^Facultad de Ciencias de La Salud, Dpto. de Ciencias Médicas Básicas, Sección Fisiología, Universidad de La Laguna, Tenerife, Spain; ^5^Institute of Psychiatry, Psychology and Neuroscience, King’s College London, London, United Kingdom

**Keywords:** functional connectivity, mental silence, resting state – fMRI, thoughless awareness, attention, mind-wandering, Sahaja yoga meditation

## Abstract

Neuroscience research has shown that meditation practices have effects on brain structure and function. However, few studies have combined information on the effects on structure and function in the same sample. Long-term daily meditation practice produces repeated activity of specific brain networks over years of practice, which may induce lasting structural and functional connectivity (FC) changes within relevant circuits. The aim of our study was therefore to identify differences in FC during the resting state between 23 Sahaja Yoga Meditation experts and 23 healthy participants without meditation experience. Seed-based FC analysis was performed departing from voxels that had shown structural differences between these same participants. The contrast of connectivity maps yielded that meditators showed increased FC between the left ventrolateral prefrontal cortex and the right dorsolateral prefrontal cortex but reduced FC between the left insula and the bilateral mid-cingulate as well as between the right angular gyrus and the bilateral precuneus/cuneus cortices. It thus appears that long-term meditation practice increases direct FC between ventral and dorsal frontal regions within brain networks related to attention and cognitive control and decreases FC between regions of these networks and areas of the default mode network.

## Introduction

Meditation involves many different contemplative practices. The general objective of most common meditation practices in western countries is to increase consciousness and harmony and reduce stress, among many other things. Different meditation techniques utilize techniques involving self-awareness, attentional focusing, emotional self-perception, etc. (Rubia, 2009). Given that meditation changes consciousness and awareness, it has raised the interest of neuroscience research on its brain effects. Brain research has found associations between the experience achieved with meditation practices and structural and functional brain changes in longitudinal studies and/or differences in brain structure and function between expert meditators compared to novices or non-meditators in cross-sectional studies [see reviews (Fox et al., 2014, 2016)]. Sahaja Yoga Meditation (SYM) is particularly interesting as a meditation technique because it teaches the practitioners to achieve the state of mental silence or thoughtless awareness, where thoughts are either suppressed or substantially reduced, which is considered the ultimate goal of meditation as mentioned in early yoga manuscripts (Kokodoko, 2014). In this study, we focused on a comparison of functional connectivity (FC) patterns at resting state (RS) between long-term Sahaja Yoga expert meditators and non-meditators based on brain regions that differed between them in gray matter volume.

While very few studies have investigated the brain structure and function effects of long-term SYM practice by means of MRI techniques, a bigger number of studies have investigated SYM effects on brain function by means of electroencephalography (EEG). The first study by Panjawani (Panjwani et al., 1996) showed that seizure reduction in patients with idiopathic epilepsy after 6 months of SYM was associated with increased ratios of EEG powers in delta, theta, alpha, and beta bands. Aftanas and Golocheikine showed that long-term SYM was characterized by increased theta synchronization between prefrontal and posterior association cortices along with less pronounced intra- and interhemispheric coherence over posterior brain regions (Aftanas and Golocheikine, 2001,2002a,b). They also showed indications of a reduction in chaotic complexity in EEG measures over midline frontal and central regions, an indicator of a reduction in the activity of the default mode network (DMN; Aftanas and Golocheikine, 2002b). Later on, Reva reported influences of SYM on event-related potentials sensitive to improvements in emotion processing (Reva et al., 2014). Our own research showed structural and functional differences in long-term practitioners of SYM relative to healthy non-meditators overlapping in the right inferior frontal cortex/insula, anterior cingulate cortex, and temporal cortex (Hernández et al., 2016, 2018, 2020), with structural differences extending further to the left inferior frontal cortex and right angular gyrus (Hernández et al., 2016, 2020). A recent study by Dodich et al. (2019) found that, even after a short period (4 weeks) of SYM training, non-meditators demonstrated similar increased gray matter in the right inferior frontal cortex/insula and changes in the coherence of intrinsic brain activity in the right IFG and anterior parts of the executive control network, suggesting a direct association between SYM practice and these brain regions.

Spontaneous RS fluctuations have been studied in experts of a variety of contemplative practices. Previous reports have shown reduced FC between nodes of the DMN but increased connectivity between nodes within attentional and executive networks (Brewer et al., 2011). The connectivity between nodes within the DMN has been of main interest, given the role of the DMN in intrinsically oriented and self-referential thought processes and mind-wandering (Buckner et al., 2008), similarly to the reversal pattern of the DMN with those called “task-positive networks” related to conflict monitoring, cognitive control, and working memory, but during RS (Fox et al., 2005). Brewer et al. (2011) argued a reduction within DMN activity and connectivity, given expert meditator reduction in mind-wandering and self-referential thought while increasing consciousness of the present moment (Brewer et al., 2011). Interestingly, this effect was observed during the practice of different meditation conditions (concentration, loving kindness, and choiceless awareness) by long-term meditators in the mindfulness/insight tradition besides baseline periods. Brewer et al. observed connectivity consistency across both meditation and baseline periods, suggesting that meditation practice may transform the resting-state experience into one that resembles a meditative state. Similarly, this pattern has been endorsed by other studies (Kilpatrick et al., 2011; Yang et al., 2016) and detailed by others (Scheibner et al., 2017) in the context of mindfulness meditation. Nonetheless, these effects have been never tested in expert SYM, which would extend the results of Brewer et al. to other meditative practices. In a broader sense, the reduction of mind-wandering with meditation is particularly relevant for mental health, given that mind-wandering and the associated DMN network have been found to be increased in many mental health disorders (Killingsworth and Gilbert, 2010; Bessette et al., 2018; Bozhilova et al., 2018).

Previous reports have suggested that meditation may serve to treat conditions featuring excessive impulsivity. Meditation (e.g., mindfulness meditation) has been shown to improve attention and inhibitory control which is associated with impulsivity (Valentine and Sweet, 1999; Jha et al., 2007; Rubia, 2009; Lattimore et al., 2011; Korponay et al., 2019). Meditation effects on impulsivity can be multifaceted, given that impulsivity is a multidimensional construct that involves the inability to sustain attention, inhibit prepotent urges, and wait or plan behavior (Lattimore et al., 2011). In fact, meditation has been shown to have positive effects on different psychopathologies which are characterized by different patterns of impulsivity such as substance use disorders (Verdejo-Garcia and Albein-Urios, 2021) or attention deficit hyperactivity disorder (Harrison et al., 2004; Rubia, 2009; Zhang et al., 2018). As far as we know, there is only one single study that evaluated impulsivity associated with RS connectivity after mindfulness meditation (Fahmy et al., 2019). Fahmy et al. (2019) observed that DMN connectivity was affected after mindfulness meditation in substance use disorder when compared to a usual treatment. Therefore, impulsivity-related dimensions may be affected by meditation expertise.

In this study, we explored the differences in RS FC between long-term SYM meditators and healthy controls (non-meditators). For this purpose, we selected seed regions that showed structural differences between experts in SYM and non-meditators reported in an earlier study (Hernández et al., 2016) and examined RS FC in the same participants. Given that SYM is subjectively characterized by a reduction in task-irrelevant thought processes (e.g., reduction in mind-wandering) and increased awareness of the present moment (e.g., increased attention) and extrapolating from findings of previous EEG studies and fMRI studies of SYM and other meditation practices (Aftanas and Golocheikine, 2001,2002a,b; Brewer et al., 2011; Hernández et al., 2016), we hypothesized that long-term SY meditators will show: (a) increased FC between nodes of attention and executive control networks and (b) reduced FC between DMN regions and attention and executive control regions. Furthermore, given the association between meditation and improved cognitive control and behavioral self-control (Tang et al., 2007; Tang and Posner, 2009), we hypothesized that FC differences would be associated with objective measures of self-control as assessed in a questionnaire of impulsiveness and with objective measures of cognitive control as assessed in motor and interference inhibition tasks.

## Experimental Procedures

### Participants

Forty-six right-handed, white Caucasian, healthy volunteers (21–63 years) participated in the study, 23 experts in SYM (17 females) and 23 non-meditators (17 females). The groups were matched on age, gender, and level of studies (see Table 1). The volunteers had no physical or mental illness, no history of neurological disorders, and no addiction to nicotine, alcohol, or other drugs. All the SYM experts and non-meditator volunteers had participated in three of our previous studies (Hernández et al., 2018, 2020; Dodich et al., 2019). The current analyses focuses on the resting-state FC acquisition that had not been previously reported. Our previous studies analyzed the differences in structural (Hernández et al., 2016, 2020) and FC during meditation state (Hernández et al., 2018) between those two samples. As such, the reported descriptive statistics on the participants are identical, while the resting-state fMRI dataset and analysis differences differ in terms of the analyzed MRI modality and respective phenomena.

**TABLE 1:** Demographic characteristics of the groups.

	Meditators, mean (SD)	Controls, mean (SD)	*t*(*df* = 44)	*t*(*df* = 42)	*t*(*df* = 42)	*p*-value^a^
Volunteers	23	23				
Age (years)	46.5 (11.4)	46.9 (10.9)	−0.13			0.89
Age range (years)	20.3–63.1	21.3–63.3				
Education degree, (range 0 to 6)	3.78 (1.2)	4.04 (1.36)	0.69			0.50
Height (cm)	167.0 (8.8)	167.2 (7.6)	0.09			0.93
Weight (kg)	69.5 (14.6)	71.7 (14.5)	0.53			0.60
Body mass index	24.9 (4.5)	25.5 (3.9)	0.54			0.60
BIS-11			*F*(1, 42)			
Attentional impulsivity	13.0 (2.90)	13.3 (2.69)	0.20			0.65
Motor impulsivity	11.65 (2.87)	12.43 (3.08)	0.71			0.40
Self-control impulsivity	11.30 (2.81)	9.13 (3.65)	5.81			0.02
Cognitive complexity	11.26 (2.96)	10.13 (2.05)	2.39			0.13
Perseverance	7.43 (2.04)	6.35 (2.46)	2.74			0.11
Cognitive instability	7.3 (2.22)	7.48 (2.19)	0.11			0.74
Total BIS-11 score	61.91 (8.32)	58.83 (6.27)	2.17			0.15
Go/no-go task						
Inhibition probability	78.69 (16.18)	85.11 (10.93)		1.52		0.13
Simon task						
RT interference effect	84.24 (40.69)	115.59 (57.97)			2.33	0.02
Accuracy interference effect	9.82 (9.50)	11.76 (11.18)			0.62	0.54

*^*a*^*p*-values represent group differences between meditators and controls using two-tailed independent-samples *t*-tests.*

The meditators were recruited from the local Tenerife SYM group in addition to SYM practitioners attending a seminar of SYM in Tenerife in January 2014. The controls were recruited through local and Facebook advertisements. The controls were not practicing any type of meditation or yoga when participating in the study. All participants filled in different questionnaires to evaluate their individual health status, education, and age. The meditators additionally filled in a questionnaire to register their experience in SYM, including years of practice, total hours of meditation, average time dedicated to meditation per day, and frequency of the perception of the state of mental silence (from never to several times a day). The meditators had between 5 and 26 years of experience of daily meditation practice in SYM [mean 14.1 (SD = 6.1) years], and the average time dedicated to meditation per day was 84.7 (SD = 32.2) min. Only three controls reported a minimum meditation experience of less than 6 month of practice. All the other participants of the control group had not any mediation experience. All participants signed informed consent to participate freely. The Ethics Committee of the University of La Laguna approved this study.

#### Behavioral and Neuropsychological Measures of Impulsiveness

Given the association between meditation and improved measures of impulsiveness (Tang et al., 2007; Tang and Posner, 2009), we also tested whether meditators differed from non-meditators in behavioral and neuropsychological measures of impulsiveness. For this purpose, the participants were asked to fill in the Barrat Impulsivity Scale (BIS-11), which is a behavioral measure of impulsiveness (Patton et al., 1995). The BIS-11 is a self-report questionnaire containing 30 questions and which requires the participants to answer in terms of frequency (e.g., from “rarely/never” to “almost always”). The items are scored from 1 to 4, yielding a total score and six first-order factors: attentional impulsivity, motor impulsivity, self-control, cognitive complexity, perseverance, and cognitive instability.

The neuropsychological assessment included two computerized tasks of cognitive control, motor and interference inhibition, respectively, i.e., the go–no-go task and the Simon task, taken from the adult version of the Maudsley Attention and Response task battery (Penadés et al., 2007; Rubia et al., 2007a).

##### Go/No-Go task

A measure of motor response inhibition, go–no-go requires a motor response to go stimuli and response inhibition to no-go stimuli. The task lasted for 2.30 min. The participants responded with their dominant hand. In 73.4% of trials, a spaceship (go stimulus) pointing right appeared in the center of the screen, and the participants must press the right arrow key as fast as possible. In 26.6% of trials, a blue planet (no-go stimulus) appeared in the center of the screen instead of a spaceship, and the participants must inhibit their response. The go and no-go stimuli were displayed for 300 ms, followed by a blank screen for 1,000 ms. There were 150 trials in total (110 go trials, 40 no-go trials). The dependent variable was the probability of inhibition to no-go stimuli.

##### Simon task

This task measured stimulus–response conflict resolution/interference inhibition and selective attention. In this task, airplanes pointing left or right appeared on the left- or right-hand side of the screen. The participants must press the arrow key that corresponds to the direction the airplane is pointing as fast as they could. In 72.73% of trials, the direction an airplane pointed and the side of the screen that it appeared were *congruent* (e.g., left airplane appears on the left and vice versa); the remaining 27.27% trials are *incongruent* trials (e.g., left airplane appeared on the right or right arrows on the left). Response conflict arose between iconic information (i.e., a left-hand response to a left-pointing airplane) and the predominant, incompatible spatial information (i.e., the airplane appears on the opposite side of the screen it is pointing toward, e.g., right). This conflict is typically reflected in slower reaction times to incongruent relative to congruent trials, and the difference between these trials (RT incongruent – RT congruent) is called the Simon reaction time effect (Simon and Berbaum, 1988).

Airplanes were displayed and then followed by a blank screen with an inter-stimulus interval of 1,400 ms. There were 220 trials in total, 160 congruent trials (80 left airplanes, 80 right airplanes) and 60 incongruent trials. The dependent variable is the Simon RT effect (i.e., RT incongruent – RT congruent, the Simon RT effect).

### MRI Acquisition and RS Protocol

Axially oriented functional images were obtained by a 3T Sigma HD MR scanner (GE Healthcare, Waukesha, WI, United States) using an echo-planar-imaging gradient-echo sequence and an eight-channel head coil (TR = 2,000 ms, TE = 21.6 ms, flip angle = 90°, matrix size = 64 × 64 pixels, 37 slices, 4 × 4 mm in plane resolution, spacing between slices = 4 mm, slice thickness = 4 mm, and interleaved acquisition). The head was stabilized with foam pads. The slices were aligned to the anterior commissure–posterior commissure line and covered the whole brain. Functional scanning was preceded by 18 s of dummy scans to ensure tissue steady-state magnetization. The volumes were 180, taken during each run for every participant at RS. The meditators did a second run with the same parameters used for RS but at meditation state, the results of which are not reported here. High-resolution sagittal-oriented anatomical images were also collected for anatomical reference. For this purpose, a 3D fast spoiled-gradient-recalled pulse sequence was obtained with the following parameters: TR = 8.761 ms, TE = 1.736 ms, flip angle = 12°, matrix size = 256 × 256 pixels, 0.98 × 0.98 mm in plane resolution, spacing between slices = 1 mm, and slice thickness = 1 mm.

During the RS functional scan, all the participants were explicitly instructed to close their eyes, relax, lie still, not to think of anything in particular, and not to fall asleep. Moreover, expert meditators were explicitly instructed not to meditate during the resting scan.

### Data Preprocessing

Prior to data acquisition, five scans (excluded from the analysis) were acquired to avoid magnetization equilibration effects. All the images were preprocessed using the Data Processing Assistant for Resting-State fMRI Advanced Edition toolbox, version 3.2, which is part of the Data Processing and Analysis of Brain Imaging (DPABI) toolbox, version 1.2 (Yan et al., 2016). The preprocessing steps included (1) slice timing by shifting the signal measured in each slice relative to the acquisition of the slice at the mid-point of each TR, (2) realignment using a least squares approach and a six-parameter (rigid body) spatial transformation, (3) co-registering individual structural images to the mean functional image of each subject, (4) T1 images were segmented into gray matter, white matter, and cerebrum spinal fluid using the diffeomorphic anatomical registration through exponentiated lie algebra (Ashburner, 2007), (5) spatial normalization of functional volumes by using the parameters extracted from the anatomical segmentation procedure in each subject and resampling the voxel size to 3 mm × 3 mm × 3 mm, (6) spatial smoothing with a 4-mm full-width-at-half-maximum Gaussian kernel, and (7) nuisance regression, including principal components (PC) extracted from subject-specific white matter and cerebrospinal fluid masks (five PC parameters) using a component-based noise correction method (Behzadi et al., 2007) as well as Friston 24-parameter model (six head motion parameters, six head motion parameters one time point before, and the 12 corresponding squared items; Friston et al., 1996). The component-based noise correction method procedure here consisted of detrending, variance (i.e., white matter and cerebral spinal fluid) normalization, and PC analysis according to Behzadi et al. (2007), and (Aftanas and Golocheikine, 2001) band-pass temporal filtering (0.01–0.1 Hz).

In order to quantify head motion, the frame-wise displacement (FD) of time series was computed based on Jenkinson et al. (2002) as suggested by Yan et al. (2016). The mean FD was controlled as a covariate of no interest in statistical analyses in order to reduce the potential effect of head motion. Following the criteria mentioned by the DPARSF developers (Yan et al., 2016), one control and one meditator subject were excluded because their head motion was beyond 2.0 mm and/or 2.0°.

### The Selection of Seed Regions

We selected the seed regions based on the results of a previous morphometry study with the same volunteers (Hernández et al., 2016). The clusters showing increased gray matter volume in experts of SYM compared to non-meditators were used as the seeds (see Table 2; seed regions available upon request).

**TABLE 2:** The selection of seed regions for functional connectivity analysis based on the morphometric effects of Sahaja Yoga Meditation expertise from the same sample [11].

Region	Side	Brodmann area	Cluster size, mm^3^	Peak MNI coordinates			Peak *T* value
				*x*	*y*	*z*	
AI, vmOFC	Right	13, 47	564	30	10	−15	5.02
ITG, FG	Right	20, 37	739	52	−43	−21	4.43
Angular gyrus	Right	39	476	52	−63	21	4.87
VLPFC	Left	11	240	−38	50	−14	4.33
AI	Left	13	543	−29	11	−9	4.27

*AI, anterior insula; FG, fusiform gyrus; ITG, inferior temporal gyrus; MNI, Montreal Neurological Institute; VLPFC, ventrolateral prefrontal cortex; and vmPFC, ventromedial prefrontal cortex.*

### RS FC Analyses

The analysis was carried out using functions in DPABI toolbox, version 1.2 (Yan et al., 2016). For FC, voxel-wise FC was calculated based on the predefined seed regions. Specifically, the mean time series was firstly computed for each participant by averaging the time series of all the voxels within the seed region, and then Pearson’s correlation between the mean time series of the seed region and time series of all other voxels within the whole brain was computed. The individual-level correlation map (r-map) was obtained for each subject, and subsequently, all r-maps were converted into z-maps with application of Fisher’s *r*-to-z transformation to obtain approximately normally distributed values for further statistical analyses.

We compared the RS FC maps between meditators and controls by using the “y_TTest2_Image” function in DPABI (Yan et al., 2016) to determine whether there were group differences in FC between each of the selected seed regions [i.e., left insula, left ventrolateral prefrontal cortex (VLPFC), and right angular gyrus] and other regions in the brain. In the independent *t*-tests, we controlled for age and gender. Moreover, we also regressed out the gray matter volumes of each seed correspondingly in a second analysis besides age and gender effects. The resulting connectivity maps between meditators and controls were corrected for multiple comparisons using the “y_GRF_Threshold” function in DPABI (Yan et al., 2016) based on Gaussian random field theory (GRF), with a threshold of | Z| > 2.3 (cluster-wise *p* < 0.05, GRF-corrected). Therefore, results are reported at a statistical threshold value of Z > 2.30 and an extended cluster threshold of 4,671 mm^3^.

#### Behavioral and Neuropsychological Measures of Impulsiveness

We first analyzed between-group differences in BIS-11 dimensions using multivariate analysis of variance. Second, we analyzed between-group differences in the key variables of the Simon test and go/no-go task using a two-sample *t*-test. Finally, between-group FC differences were correlated with behavioral variables showing significant differences between groups.

## Results

**FIGURE 1:**
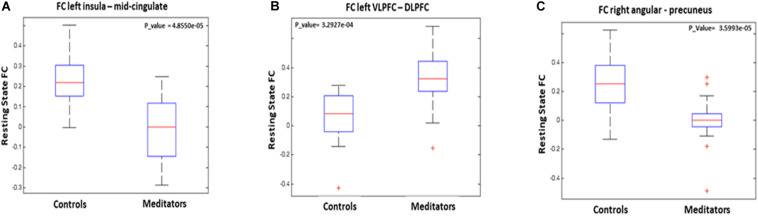
Graphical representation of functional connectivity (FC) results: **(A)** FC differences between left insula and midcingulate, **(B)** FC differences between left ventrolateral prefrontal cortex and right dorsolateral prefrontal cortex, and **(C)** FC differences between right angular gyrus adn bilateral precuneus.

**FIGURE 2:**
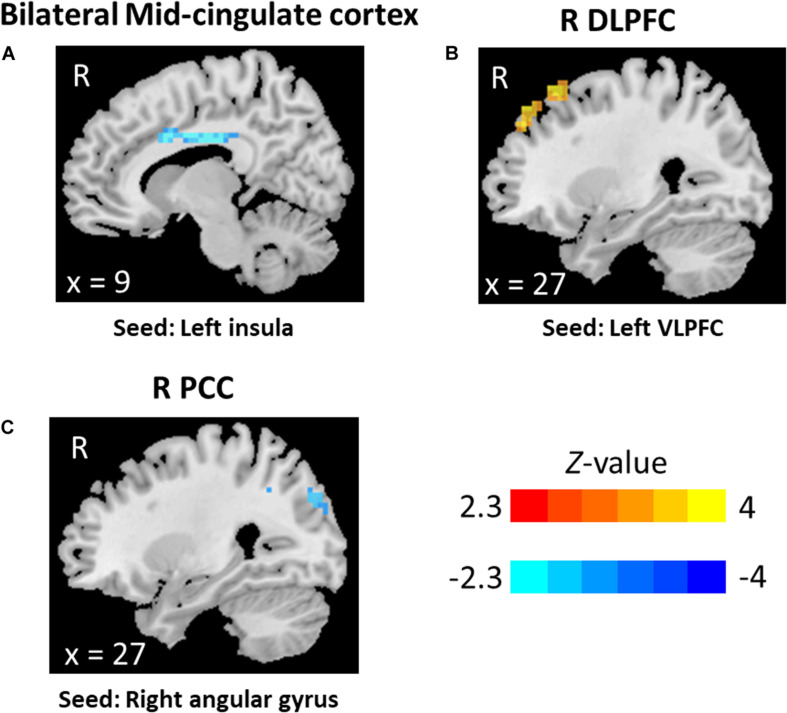
Functional connectivity results: **(A)** bilateral mid-cingulate, seed at the left insula, **(B)** right dorsolateral prefrontal cortex, seed at left ventrolateral prefrontal cortex, and **(C)** right precuneus, seed at right angular.

The statistical analyses showed significant group differences in FC when seeding at left insula, left VLPFC, and right angular gyrus (see Table 3 and Figures 1, 2). We found increased FC between left VLPFC and right dorsolateral prefrontal cortex (DLPFC) in meditators compared to controls, and there were reduced FC between the left insula and mid-cingulate cortex and between the right angular gyrus and precuneus extending to the superior occipital cortex and cuneus. However, no significant results were found when seeding at the anterior insula and inferior temporal gyrus. Finally, no significant FC differences were observed when individual gray matter volumes were regressed out. This last result can be interpreted twofold. First, given that individuals’ gray matter volume effects are already different between groups, we may be observing a collinearity effect. Second, structural effects subserve the observed functional effects. This second option should be carefully considered given the possibility of collinearity.

**TABLE 3:** Functional connectivity (FC) analyses showing resting state connectivity differences between expert meditators and controls.

Seed region	Meditators > controls	Hemisphere	Cluster size, mm^3^	Peak *T*-value^a^	MNI coordinates X Y Z
Left insula	Mid-cingulate cortex	Bilateral	4,833	–4.37	9 −12 30
Left VLPFC	DLPFC	Right	4,914	3.92	27 27 60
Right angular gyrus	Precuneus Superior occipital cortex Cuneus	Bilateral Right Right	4,671	–3.33	27 −81 36

*^*a*^Peak intensity positive or negative value informs about the positive or negative FC between regions (meditators > controls), respectively. MNI, Montreal Neurological Institute; VLPFC, ventrolateral prefrontal cortex; and DLPFC, dorsolateral prefrontal cortex.*

### Behavioral and Neuropsychological Measures of Impulsiveness

As shown in Table 1, the SYM showed an increased self-control score in the BIS-11 compared to the control participants [*F*(df = 1, 43)5.09, *p* = 0.03], but there were no significant differences in any of the other subscales. In the neuropsychological measures, two outliers were excluded in the go/no-go task, and two other different ones were excluded in the Simon task after Mahalanobis distance (*p* < 0.001). There were no differences between groups in the go/no-go task performance (*p* > 0.1). SYM showed a reduced Simon interference reaction time effect relative to the non-meditators [*t*(41) = 2.33; *p* = 0.04].

Finally, we correlated the FC between those regions which had shown significant differences between groups with Simon RT interference. The results showed that Simon RT interference was significantly correlated with the FC between the left insula and the mid-cingulate in the SYM group [*r*(21) = -0.46; *p* = 0.03]. This correlation was significantly different (z-fisher = 2.42; *p* = 0.01) to the one in the control group [*r*(21) = 0.30; *p* = 0.2].

## Discussion

### Increased FC in Meditators Relative to Non-Meditators

Experienced meditators relative to non-meditators showed increased FC between the left VLPFC and the right DLPFC, two regions related to cognitive control and conflict resolution processes and also important attention regions of the ventral (VLPFC) and dorsal (DLPFC) attention networks, respectively, (Vossel et al., 2014; Hung et al., 2018; Ryman et al., 2019). These findings suggest that long-term meditation is associated with a strengthening of dorsal and ventral attention and executive control networks. We furthermore also found a strengthening of the anticorrelation with other parts of the ventral attention networks such as insula and angular gyrus and regions of the DMN, such as anterior cingulate cortex and precuneus.

Abnormalities in FC between attentional and executive networks like DLPFC and VLPFC as well as reduced anticorrelation between regions related to attention/executive control and regions of the DMN have commonly been associated with cognitive and affective mental health disorders such as autism, depression, obsessive–compulsive disorder, and attention deficit hyperactivity disorder (Rubia et al., 2014; Sripada et al., 2014; He et al., 2016). The DLPFC is an important region for executive functions such as conflict resolution, cognitive control, and working memory and forms part of the dorsal attentional network (Corbetta and Shulman, 2002; Dosenbach et al., 2007). The left VLPFC is not only part of the ventral attention system (Jha et al., 2007) but also an important executive function region for inhibitory self-control (Rubia et al., 2007b, 2010; Hampshire et al., 2010; Zhang and Li, 2012). Thus, bilateral VLPFC has been associated with inhibitory control in fMRI (Rubia et al., 2003; Aron and Poldrack, 2006), lesions (Aron et al., 2003; Juan and Muggleton, 2012), and transcranial magnetic stimulation studies (Chambers et al., 2007; Juan and Muggleton, 2012). The bilateral VLPFC and inferior parietal regions are also part of the ventral attention system and are known to mediate attention allocation to behaviorally relevant salient stimuli (Corbetta et al., 2008; Shulman et al., 2009) and hence reflect top–down-orienting attentional processes that interact with, expedite, and underlie good inhibitory self-control (Duann et al., 2009; Hampshire et al., 2010). Collectively, as long-term meditation experience can be considered as attention training (Rubia, 2009), the enhanced connectivity between these regions may reflect improved cognitive control for a conscious experience through attention regulation.

The findings of improved FC in areas of self-control and cognitive control are further reinforced by the behavioral findings of improved self-control in SYM *versus* controls in the objective measures of the BIS-11 and of superior performance in the SIMON interference inhibition task. Inhibitory self-control and interference inhibition have been shown to be improved with meditation (Tang et al., 2007; Rubia, 2009; Tang and Posner, 2009) and, as discussed above, are mediated by VLPFC and DLPFC. Furthermore, there is evidence that better anti-correlation between the DMN and cognitive control networks is associated with better performance in cognitive control tasks and less attentional lapses (Raichle et al., 2001).

While we found increased connectivity between left VLFPC and right DLPFC in SYM experts, Hasenkamp and Barsalou observed a pattern of increased FC between a region in the right DLPFC and ipsilateral portions of insula and VLPFC (Hasenkamp and Barsalou, 2012; Hasenkamp et al., 2012). This discrepancy may be related to the involvement of different attentional and cognitive control networks depending on the meditation practice (Brewer et al., 2011). SYM shares some common goals and experiences with other meditations like mindfulness or several Buddhist traditions like Shamatha, Vipassana, or other Tibetan-style Buddhist meditations included in Hansenkamp and Barsalou (Hasenkamp and Barsalou, 2012). Importantly, a common goal between SYM and these other meditation techniques is to keep the attention in the here and now at the present moment, detecting and correcting mind-wandering episodes. However, while some of these other meditation techniques have, as a key attentional focus, their own breathing (Hasenkamp and Barsalou, 2012), SYM is based on the spontaneous (Sahaja = spontaneous) awakening of the Kundalini energy (Coward, 1985), which meditators subjectively perceive as a cool breeze when they put their hands some centimeters above their head, which is associated with the achievement of yoga (yoga = union; Devi, 1997; Hendriks, 2018; Hernández et al., 2020). The “kundalini awakening” is suggested to allow the practitioners to perceive the state of their “subtle centers” called chakras that SYM practitioners mention that they perceive through “reflex points” that the chakras have in their hands (Devi, 1997; Morgan, 1999). It is possible that these experiences, which are specific to SYM, may be related to the differences here described.

### Reduced FC in Meditators Relative to Non-Meditators

Precuneus and midcingulate form part of the posterior and anterior nodes of the DMN, respectively, (Raichle et al., 2001; Utevsky et al., 2014). The reduced FC in meditators compared to non-meditators between left insula and right angular gyrus and these areas of the DMN could suggest that long-term meditation practice improves the anticorrelation between brain regions that are commonly associated with meditation such as the insula, important for interoceptive perception (Sagliano et al., 2019) and part of the saliency network (Chong et al., 2017) and the DMN. Similarly, the angular gyrus is a key part of the ventral attention network (Heinen et al., 2017; Igelström and Graziano, 2017), and its decreased connectivity with precuneus, part of the DMN, could reflect a more mature anticorrelation between ventral attention and DMN networks.

The anticorrelation between regions of the ventral attention networks and regions of the DMN is likely to reflect the ability to switch off mind-wandering during cognitive tasks, which has been associated with better cognition and attention (Hasenkamp et al., 2012; Kane and McVay, 2012; Randall et al., 2014). This anticorrelation between attention and DMN networks increases with age in normal development (Sripada et al., 2014) and has been associated with greater maturity and better mental health, given that both younger populations and psychiatric or neurological patients typically suffer from increased DMN interference and worse anticorrelation between attention/executive and DMN networks (Sripada et al., 2014; Esposito et al., 2018). It is thus plausible that the anticorrelation between attentional and DMN regions in long-term meditators is related to the daily practice of switching off irrelevant thinking during the meditation practice (Rubia, 2009).

This anticorrelation is also in line with findings from other meditation practices where reduced RS FC was observed between nodes of the DMN and attentional/executive nodes in relation to the meditation experience (Pagnoni and Cekic, 2007; Brewer et al., 2011; Pagnoni, 2012). Similarly, Berkovich-Ohana showed reduced connectivity between the angular gyrus and the precuneus within the DMN in expert mindfulness meditators (Berkovich-Ohana et al., 2016). As described by these previous studies, we suggest that this effect may be related to changes in inner cognition and self-referential processing of expert meditators. Meditators with daily practice subjectively report changes of their focus of attention to the present moment, keeping a certain distance and observation of thoughts and emotions, as well as the ability to observe and reduce their own feelings and thoughts to be conscious about their current experience. All of these are arguably the opposite to DMN-related random mind-wandering functioning. Importantly, SYM practice teaches the practitioners to achieve the state of mental silence, which is a state with reduced or no thoughts. This repeated process of achieving a reduction in thoughts likely leads to reduced mental clutter and mind-wandering, hence switching off interferences from the DMN. The current results extend previous findings of EEG studies by Aftanas and Golocheikine with practitioners of SYM, which demonstrated reduced chaotic complexity in EEG signals in meditators relative to novices, thought to reflect reduced mind-wandering or mental clutter (Aftanas and Golocheikine, 2002a,b). Furthermore, we observed that FC between the left insula and the midcingulate was correlated with interference inhibition during the Simon task (Simon RT interference). The anterior insula and cingulate cortex are central nodes in cognitive control and conflict resolution that had shown a reduced pattern of activation in fMRI conflict task in expert meditators and changes in short-term meditators (Kozasa et al., 2018). In our study, the connectivity between these areas was shown to be reduced in SYM. Other reports had likewise shown reduced time to resolve conflict after short-term meditation training (Tang et al., 2007, 2009; Fan et al., 2015). Our results extend previous reports on conflict resolution speed into long-term SYM practitioners, particularly related to a pattern of increased connectivity between the anterior insula and the midcingulate. The midcingulate is a key area of cognitive control and conflict detection (Botvinick et al., 1999, 2001; Carter et al., 2000; Braver, 2001; Ridderinkhof et al., 2004), which is typically co-activated during conflict with insular regions that are part of visual attention networks (Walsh et al., 2011). After conflict detection, the midcingulate is also thought to exert top-down influence on other brain structures such as the prefrontal regions to adjust future performance (Botvinick et al., 2001; Danielmeier et al., 2011). The stronger FC between key areas of cognitive control/conflict detection and attention control in SYM was thus associated with better performance in a cognitive control task that requires conflict detection and attention control, suggesting that the brain connectivity benefits were associated with behavioral advantage in cognitive and attention control.

In sum, these results show that long-term SYM practice has important effects on FC, enhancing frontal attention and cognitive control networks, increasing the anti-correlation between attention and DMN networks, and improving self-control and cognitive inhibition. Moreover, the overlap with the long-term structural SYM effects indicates that the FC differences between groups are driven substantially by an underlying anatomical difference between groups rather than solely by a true metabolic difference.

### Limitations of the Study

Despite implicit instructions to meditators not to enter into a meditation state during the RS, it is possible that long-term meditators, due to their long-term practice, would naturally have reduced mind-wandering and mental clutter during rest and enter a semi-meditative state, which could have led to the connectivity differences compared to controls. Furthermore, the overlap of causes for FC and structural differences led to ambiguity in data interpretation. In our study, we removed the contribution of structural gray matter volume from FC analyses, and the FC differences disappeared. This result may be explained by the gray matter volume inhomogeneities across subjects and the correlation of their variation to modeled parameters, and apparently, the FC differences are likely due to underlying tissue differences (Oakes et al., 2007). Future studies studying the FC effects of SYM may define these FC seeds in order to test for their effects on a different sample.

## Conclusion

In conclusion, in this study, we found that a long-term expertise in SYM was associated with FC changes in the brain when compared to healthy controls without meditation experience. The SYM experts showed a pattern of increased connectivity between nodes in frontal attentional and executive networks at RS. Similarly, brain regions from task positive networks in the insula and the parietal attention regions showed an increased anticorrelation with medial regions of the DMN. These results might be suggestive of a strengthening of attention and executive control networks and a weakening of mind-wandering. Finally, we would like to highlight three most interesting contributions of our research. First, it is the first time SYM experts have been studied during resting-state fMRI. The findings will hence serve to extend the previous findings in FC studying other meditation techniques (Tomasino et al., 2013). Second, this study provides evidence of a difference between meditators and non-meditators in FC at RS based on seeds already showing structural alterations in the same sample, thus linking structural and FC findings. Third, FC differences between groups were shown to be associated to performance in a neuropsychological test, making a valuable contribution to clarify the mechanisms of SYM.

## Data Availability Statement

The raw data supporting the conclusions of this article will be made available by the authors, without undue reservation.

## Ethics Statement

The studies involving human participants were reviewed and approved by Ethics Committee from the Universidad de La Laguna. The patients/participants provided their written informed consent to participate in this study.

## Author Contributions

AB-L: participated in experimental design, data acquisition, data analysis and manuscript writing, and critical review. SH: participated in experimental design, data acquisition, data analysis, manuscript writing, and critical review. YX: participated in data analysis, manuscript writing, and critical review. JG-M: participated in experimental design, data acquisition, and manuscript’s critical review. KR: participated in experimental design, manuscript writing, and critical review. All authors contributed to the article and approved the submitted version.

## Conflict of Interest

KR has received funding from TAKEDA pharmaceuticals for another project. The remaining authors declare that the research was conducted in the absence of any commercial or financial relationships that could be construed as a potential conflict of interest.
